# ﻿Updated taxonomy on *Gerronema* (Porotheleaceae, Agaricales) with three new taxa and one new record from China

**DOI:** 10.3897/mycokeys.89.79864

**Published:** 2022-04-29

**Authors:** Qin Na, Yaping Hu, Hui Zeng, Zhizhong Song, Hui Ding, Xianhao Cheng, Yupeng Ge

**Affiliations:** 1 Shandong Key Laboratory of Edible Mushroom Technology, School of Agriculture, Ludong University, Yantai 264025, China Ludong University Yantai China; 2 Nanjing Institute of Environmental Sciences, Ministry of Ecology and Environment, State Environmental Protection Scientific Observation and Research Station for Ecological Environment of Wuyi Mountains, 8 Jiangwangmiao street, Nanjing 210042, China Nanjing Institute of Environmental Sciences, Ministry of Ecology and Environment Nanjing China; 3 Institute of Edible Fungi, Fujian Academy of Agricultural Sciences; National and Local Joint Engineering Research Center for Breeding & Cultivation of Features Edible Fungi, Fuzhou 350014, China Fujian Academy of Agricultural Sciences; National and Local Joint Engineering Research Center for Breeding & Cultivation of Features Edible Fungi Fuzhou China

**Keywords:** new taxon, polygenes, taxonomy, white-spored

## Abstract

Only three *Gerronema* (Porotheleaceae) species have been previously recorded in China. Here, we report collections of a fourth species in China: *G.nemorale* Har. Takah., which is widely distributed in Chinese temperate to subtropical zones. We also formally describe three new species, collected from Anhui, Fujian, and Zhejiang provinces: *G.baishanzuense***sp. nov.**, *G.microcarpum***sp. nov.**, and *G.zhujian***sp. nov.** Furthermore, we include the results of a phylogenetic analysis of Porotheleaceae based on a multi-locus (ITS + nLSU) dataset. The results, which indicate that *Gerronema* is polyphyletic, support the taxonomic recognition of the three new species. Morphological descriptions, photographs, line drawings, and comparisons with closely related taxa are presented for the new and newly recorded species. A key to the seven species of *Gerronema* in China is also provided.

## ﻿Introduction

*Gerronema* Singer is a small omphalinoid genus, principally subtropical to tropical in distribution, with approximately 62 named species in Index Fungorum. Singer (1951) erected the genus *Gerronema* to accommodate three tenacious and lignicolous omphalinoid to clitocyboid species from South America and later transferred some species traditionally placed in *Omphalina* Quél. to this new genus (Singer 1964). This taxonomic definition of *Gerronema* was controversial, however, as the circumscription of *Omphalina* by Singer was notably different from that of Bigelow (Bigelow 1970; Singer 1986). Virtually all species of *Omphalina* recognized by Bigelow were included in Singer’s concept of *Gerronema*, whereas species placed in *Clitocybe* (Fr.) Staude by Bigelow were retained in *Omphalina* by Singer (Bigelow 1970, 1982, 1985; Singer 1986). Furthermore, Lange (1981) treated *Gerronema* as a subgenus of Omphalina (subgen. Gerronema). Both Singer and Bigelow considered *Gerronema* to be pigment based and therefore emphasized pigmentation as a more important taxonomic character than other observed features (Bigelow 1970, 1982, 1985; Singer 1986). *Gerronemasensu* Singer, however, was considered to be heterogeneous (Clémençon 1982; Moser 1983; Kuyper 1986; Singer 1964, 1975, 1986; Norvell et al. 1994; Antonín et al. 2008), and Redhead (1986) restricted the genus to species having sarcodimitic tissues, a concept supported by Norvell et al. (1994). Along with Redhead, Norvell et al. defined *Gerronema* as comprising lignicolous species with typical sarcodimitic tissues, and the genus was monophyletic according to this circumscription (Redhead 1986; Norvell et al. 1994). Finally, an infrageneric classification proposed by Singer divided *Gerronema* into four subgenera containing six sections on the basis of pigmentation, cystidia, hymenophoral trama, and clamp connections (Singer 1970).

In previous taxonomic studies, many authors have suggested that the genus *Gerronema* is heterogeneous (Clémençon 1982; Moser 1983; Kuyper 1986; Antonín et al. 2008). The polyphyletic status of *Gerronema* is uncertain, however, owing to insufficient species representation and limited phylogenetic evidence, and only four *Gerronema* taxa have been analyzed in phylogenetic studies: *G.chrysophyllum* (Fr.) Singer, *G.strombodes* (Berk. & Mont.) Singer, *G.subclavatum* (Peck) Singer ex Redhead, and *G.marchantiae* Singer & Clémençon (Lutzoni 1997; Pine et al. 1999; Hibbett and Binder 2002; Moncalvo et al. 2002; Redhead et al. 2002). Two of these species, *G.chrysophyllum* and *G.marchantiae*, have since been transferred to *Chrysomphalina* Clémençon (Clémençon 1982) and *Loreleia* Redhead, Moncalvo, Vilgalys & Lutzoni (Redhead et al. 2002), respectively, and the other two species, *G.subclavatum* and *G.nemorale*, are difficult to distinguish genetically (Antonín et al. 2008). According to a phylogenetic reconstruction of more than 800 euagaric taxa derived from a nuclear ribosomal large subunit RNA gene (nLSU) sequence dataset, *Gerronema* is monophyletic and belongs to the “hydropoid” clade together with *Hydropus* Kühner ex Singer *s. str.*, *Megacollybia* Kotl. & Pouzar, *Clitocybula* (Singer) Singer ex Métrod, and *Porotheleumfimbriatum* (Pers.) Fr. (Moncalvo et al. 2002). Matheny et al. (2006) and Antonín et al. (2019) concurred with Moncalvo et al. (2002) in the establishment of the hydropoid group and the monophyly of *Gerronema*. Matheny et al. (2006) also included *Henningsomycescandidus* (Pers.) Kuntze, *Hydnopolyporusfimbriatus* (Cooke) D.A. Reid, and some *Mycena* species (*M.auricoma* Har. Takah., *M.amabilissima* (Peck) Sacc. and *M.aurantiidisca* (Murrill) Murrill) in the same subclade of the large Marasmioid clade, but they did not include any *Gerronema* species in their studies. In a taxonomic and phylogenetic study of *Clitocybulas. l.*, the hydropoid clade was found to comprise eight genera, including *Gerronema*, and was sister to other genera (Antonín et al. 2019). In 2019, Vizzini et al. assigned the hydropoid clade to Porotheleaceae, a new family; at the same time, they recognized *Gerronema* as polyphyletic on the basis of previous taxonomic studies and divided it into seven clades, designated as *Gerronema* 1 to *Gerronema* 7 (Vizzini et al. 2019). The view of *Gerronema* as polyphyletic is also supported by our studies.

*Gerronema* is well characterized by its lignicolous habit; omphalinoid to clitocyboid basidiomata; an umbonate or infundibuliform pileus with partly to entirely pigmented, decurrent lamellae; smooth, thin-walled, and inamyloid basidiospores; cystidia that are present or absent; and sarcodimitic tramal tissues (Singer 1951; Norvell et al. 1994). Species of *Gerronema* are widespread in subtropical to tropical regions but are also rarely distributed in temperate zones (Singer 1951, 1970; Norvell et al. 1994). Studies of *Gerronema* during the past 70 years have focused on species distributed in South America and Asia, with 11 new species and six new combinations recognized from Argentina, the USA, Brazil, Japan, India and China (Singer 1951, 1959; Liu 1995; Takahashi 2000; Desjardin et al. 2005; Latha et al. 2018; Liu et al. 2019). In contrast, few investigations of *Gerronema* taxa in Europe, Australia, and Africa have been conducted, and only one new species and two new combinations have been reported from these regions (Bañares et al. 2006; Degreef and Ndong 2007; Cooper 2014).

Only three *Gerronema* species, including two new to the genus, have previously been recognized in China (Liu 1995; Dai et al. 2010; Liu et al. 2019). In recent years, progress has been achieved in clarifying the status of mycenoid and omphalinoid fungi in China, including a new taxon reported from Zhejiang Province, China, namely, *Leucoinocybelishuiensis* Q. Na, H. Zeng & Y.P. Ge, which is sister to *Gerronema* (Na and Bau 2018, 2019a, 2019b; Ge et al. 2021; Na et al. 2021). During our ongoing research on omphalinoid fungi, we discovered three new species belonging to *Gerronema* in subtropical China. These species are formally described here as *G.baishanzuense* Q. Na, H. Zeng & Y.P. Ge, *G.microcarpum* Q. Na, H. Zeng & Y.P. Ge, and *G.zhujian* Q. Na, H. Zeng & Y.P. Ge. In addition, we have determined that *G.nemorale* Har. Takah., which has not previously been recorded in China, is widely distributed in the country. We accordingly present a morphological description of the new and newly recorded species, and we also provide a key for identification of the seven species of *Gerronema* currently known from China.

## ﻿Materials and methods

### ﻿Sample collection and morphological description

Specimens were collected in Anhui, Fujian, Jilin, and Zhejiang provinces, China, from June 2019 to August 2021. Basidiomata were photographed in their natural habitats with a Canon 90D digital camera and then dried on allochroic silica gel. Fresh fruiting bodies were recorded in the field to identify macroscopic characters. In our descriptions, color codes and notations follow Kornerup & Wanscher (Kornerup and Wanscher 1978). Microscopic features were conducted on dried specimens mounted in 5% KOH and stained with Congo red when necessary. Melzer’s reagent was used to test whether spores and tissues were amyloid (Horak 2005). Twenty mature basidiospores from each basidiocarp were measured, the notation [*a*/*b*/*c*] used at the beginning of each basidiospore description indicates that *a* basidiospores from *b* basidiocarps of *c* specimens were measured. The dimensions of basidiospores and *Q* values are presented as (d) e–f–g (h) × (i) j–k–l (m), where *d* is the minimum length, *e*–*g* represents the range of at least 90% of values, f is the average length, and *h* is the maximum length; width (*i*–*m*) is expressed in the same manner. In addition, Q is the length: width ratio of a spore, and Q ± SD is the average Q of all basidiospores ± the sample standard deviation (Ge et al. 2021; Liu et al. 2021; Na et al. 2021). Hyphae of the pileipellis and stipitipellis and a total of 20 basidia, cheilocystidia, and caulocystidia were measured from each collection. Author abbreviations follow those used in Index Fungorum (https://www.indexfungorum.org). Voucher specimens have been deposited in the Fungarium of the Fujian Academy of Agricultural Sciences (FFAAS), China.

### ﻿Phylogenetic reconstruction

Genomic DNA was extracted from dried specimens using a NuClean Plant Genomic DNA kit (Kangwei Century Biotechnology Co., Beijing, China). The internal transcribed spacer (ITS) region and the nuclear large subunit (nLSU) of ribosomal DNA were respectively amplified with primer pairs ITS1/ITS4 and LR0R/LR7 (White et al. 1990; Hopple and Vilgalys 1999). The PCR thermocycling protocol (for both ITS and nLSU) was the same as reported in Ge et al. (2021). A dataset comprising sequences from 38 accessions of seven genera of Porotheleaceae and *Mycenapurpureofusca* as an outgroup was compiled for phylogenetic analysis. All newly generated sequences and those downloaded from GenBank are shown in Table 1. The sequences used in this study were aligned and adjusted manually using BioEdit 7.0.4.1 and Clustal X (Thompson et al. 1997; Hall 1999). In the alignment, gaps were treated as missing data. The alignment was deposited in TreeBase (submission ID: 29143; study accession URL: http://purl.org/phylo/treebase/phylows/study/TB2:S29143). The best model of nucleotide evolution for the ITS and nLSU data was identified using Modeltest 2.3 (Nylander 2004). The optimized sequence dataset was analyzed using Bayesian inference (BI) and maximum likelihood (ML) methods in MrBayes 3.2.6 and raxmlGUI 1.5b1, respectively (Ronquist and Huelsenbeck 2003; Stamatakis 2006). The BI analysis was performed for 2 million generations, with trees sampled every 100 generations. The sampled trees were subsequently summarized by using the “sump” and “sumt” commands after discarding the first 25% of iterations as burn-in. For the ML analysis, default parameters in RAxML were used with 1,000 bootstrap replicates. Phylogenetic trees were visualized with Figtree 1.4.3.

**Table 1.** Sequenced specimens used in phylogenetic analysis. New and newly recorded species are marked in bold.

No.	Taxa	Voucher	Locality	ITS Sequences ID	nLSU Sequences ID	Reference
1	*Chrysomycenaperplexa*	MCVE:30184	Italy	MN496427	NG071251	Vizzini et al. 2019
2	*Clitocybulaabundans*	STU:SMNS-B-FU-2017/00898	not indicated	MF627833	–	from GenBank
3	*C.familia*	PRM 921866	Czech	JF730327	JF730320	Antonín et al. 2011
4	*C.familia*	BRNM 736053	Slovakia	JF730328	JF730323	Antonín et al. 2011
5	*C.familia*	2319-QFB-25741	not indicated	KM406970	–	from GenBank
6	*C.familia*	STU:SMNS-B-FU-2017/00926	not indicated	MF627834	–	from GenBank
7	*C.familia*	NAMA 2017-349	not indicated	MH979253	–	from GenBank
8	*C.flavoaurantia*	D	Italy	HM191743	–	Malysheva and Morozova 2011
9	*C.flavoaurantia*	GDOR	Italy	HM191744	–	Malysheva and Morozova 2011
10	*C.flavoaurantia*	LE 262757	Russia	HM191745	–	Malysheva and Morozova 2011
11	*C.lacerata*	LE 6639	Russia	HM191746	–	Malysheva and Morozova 2011
12	*C.lacerata*	LE 262744	Russia	HM191747	–	Malysheva and Morozova 2011
13	*C.lacerata*	LE 262743	Russia	HM191748	–	Malysheva and Morozova 2011
14	*C.lignicola*	BPI M-20.989	Russia	HM191735	–	Malysheva and Morozova 2011
15	*C.lignicola*	BPI M-20.825	Russia	HM191736	–	Malysheva and Morozova 2011
16	*C.lignicola*	LE253926	Russia	HM191741	–	Malysheva and Morozova 2011
17	*C.lignicola*	LE262737	Russia	HM191742	–	Malysheva and Morozova 2011
18	*C.oculus*	AFTOL-ID 1554	USA	DQ192178	–	Matheny et al. 2006
19	*C.oculus*	3512	not indicated	KM406971	–	from GenBank
20	*C.oculus*	BIOUG24046-B03	Canada	KT695321	–	Telfer et al. 2015
21	*C.oculus*	WU 20008	Austria	LT854017	LT854017	Antonín et al. 2019
22	*C.oculus*	S.D. Russell iNaturalist # 8591258	India	MN906164	–	from GenBank
23	*C.oculus*	S.D. Russell iNaturalist # 8606755	India	MN906165	–	from GenBank
24	*Gerronemaatrialbum*	AFTOL-ID 1529	USA	DQ192179	DQ192179	Matheny et al. 2006
25	***G.baishanzuense***	**FFAAS0359 Holotype**	**China**	**OL985962**	**OL985984**	**This study**
26	***G.baishanzuense***	**FFAAS0360**	**China**	**OL985963**	–	**This study**
27	***G.baishanzuense***	**FFAAS0361**	**China**	**OL985964**	**OL985985**	**This study**
28	***G.baishanzuense***	**FFAAS0362**	**China**	**OL985965**	**OL985986**	**This study**
29	***G.baishanzuense***	**FFAAS0363**	**China**	**OL985966**	**OL985987**	**This study**
30	***G.baishanzuense***	**FFAAS0366**	**China**	**OL985967**	**OL985988**	**This study**
31	*G.indigoticum*	HMJAU 47636	China	MK693727	MK693732	Liu et al. 2019
32	*G.indigoticum*	HMJAU 47942	China	MK693728	MK693733	Liu et al. 2019
33	*G.indigoticum*	HMJAU 47943	China	MK693729	MK693734	Liu et al. 2019
34	*G.keralense*	CAL 1666	India	MH156555	NG_064531	Latha et al. 2018
35	*G.kuruvense*	CAL 1665	India	NG_159831	NG_064530	Latha et al. 2018
36	***G.microcarpum***	**FFAAS036**5	**China**	–	**OL985989**	from GenBank
37	***G.microcarpum***	**FFAAS0371**	**China**	**OL985968**	**OL985990**	from GenBank
38	***G.microcarpum***	**FFAAS0372**	**China**	**OL985969**	**OL985991**	from GenBank
39	***G.microcarpum***	**FFAAS0373 Holotype**	**China**	**OL985970**	**OL985992**	from GenBank
40	***G.microcarpum***	**FFAAS0374**	**China**	**OL985971**	–	from GenBank
41	***G.microcarpum***	**FFAAS0375**	**China**	**OL985972**	**OL985993**	from GenBank
42	*G.nemorale*	KACC 43599	Korea	EU883592	–	**This study**
43	*G.nemorale*	KACC 43600	Korea	EU883593	–	**This study**
44	*G.nemorale*	not indicated	Korea	EU883594	–	**This study**
45	*G.nemorale*	FA249	Pakistan	MN744686	–	**This study**
46	*G.nemorale*	FA236	Pakistan	MN744687	–	**This study**
47	*G.nemorale*	FA239	Pakistan	MN744688	–	**This study**
48	***G.nemorale***	**FFAAS0377**	**China**	**OL985976**	**OL985997**	**This study**
49	***G.nemorale***	**FFAAS0379**	**China**	**OL985977**	**OL985998**	**This study**
50	***G.nemorale***	**FFAAS0382**	**China**	**OL985978**	**OL985999**	**This study**
51	***G.nemorale***	**FFAAS0384**	**China**	**OL985979**	**OL986000**	**This study**
52	***G.nemorale***	**FFAAS0388**	**China**	**OL985980**	**OL986001**	**This study**
53	***G.nemorale***	**FFAAS0389**	**China**	**OL985981**	**OL986002**	**This study**
54	***G.nemorale***	**FFAAS0392**	**China**	**OL985982**	**OL986003**	**This study**
55	***G.nemorale***	**FFAAS0410**	**China**	**OL985983**	**OL986004**	**This study**
56	*G.strombodes*	DJL05NC72	USA	EU623639	–	Hughes et al. 2007
57	*G.strombodes*	TFB12519/TENN60718	USA	EU623640	–	Hughes et al. 2007
58	*G.strombodes*	TFB12783/TENN61350	USA	EU623641	–	Hughes et al. 2007
59	*G.strombodes*	TFB11947 clone C2	USA	KY242503	–	Hughes et al. 2007
60	*G.strombodes*	TFB11947 clone C3	USA	KY242504	–	Hughes et al. 2007
61	*G.strombodes*	TFB11947 clone C5	USA	KY242506	–	Hughes et al. 2007
62	*G.strombodes*	TFB14234	USA	KY242507	–	Hughes et al. 2007
63	*G.strombodes*	TFB14514	USA	KY242509	–	Hughes et al. 2007
64	*G.strombodes*	TFB11947	USA	KY271083	–	from GenBank
65	*G.subclavatum*	Redhead 5175, DAOM	not indicated	U66434	–	Lutzoni 1997
66	*G.subclavatum*	FLAS-F-60986	USA	MH016932	–	from GenBank
67	*G.subclavatum*	FLAS-F-61518	USA	MH211945	–	from GenBank
68	*G.subclavatum*	Smith-2018	USA	MK573888	–	Direct Submission
69	*G.subclavatum*	Mushroom Observer # 243440	USA	MK607510	–	Direct Submission
70	*G.subclavatum*	iNaturalist # 8545787	India	MN906021	–	from GenBank
71	*G.subclavatum*	S.D. Russell MycoMap # 6854	India	MN906138	–	from GenBank
72	*G.viridilucens*	SP307883 (SP)	Brazil	–	EF514207	Desjardin et al. 2005
73	*G.waikanaense*	PDD:87667	New Zealand	JQ694117	–	from GenBank
74	*G.wildpretii*	BRNM 788347	Madeira	LT854045	LT854043	Antonín et al. 2019
75	*G.xanthophyllum*	PRM 924657	Czech	LT854023	LT854023	Antonín et al. 2019
76	***G.zhujian***	**FFAAS0364**	**China**	**OL985973**	**OL985994**	**This study**
77	***G.zhujian***	**FFAAS0370**	**China**	**OL985974**	**OL985995**	**This study**
78	***G.zhujian***	**FFAAS0376 Holotype**	**China**	**OL985975**	**OL985996**	**This study**
79	*Hydropusfuliginarius*	DAOM196062	USA	–	AF261368	Moncalvo et al. 2002
80	*H.marginellus*	AFTOL-ID 1720	Czech	DQ490627	DQ457674	Matheny et al. 2006
81	*H.marginellus*	OSC 112834	USA	EU669314	EU852808	from GenBank
82	*Leucoinocybelishuiensis*	FFAAS 0111	China	MW424488	MW424492	Na et al. 2021
83	*L.lishuiensis*	FFAAS 0112	China	MW424489	MW424493	Na et al. 2021
84	*L.lishuiensis*	FFAAS 0113	China	MW424490	MW424494	Na et al. 2021
85	*L.lishuiensis*	FFAAS 0115	China	MW424491	MW424495	Na et al. 2021
86	*L. sp.*	KA12-0435	South Korea	KR673482	–	Kim et al. 2015
87	*L.sulcata*	CAL 1246 (HOLOTYPE)	India	KR029720	KR029721	Latha et al. 2015
88	*L.taniae*	BCN-SCM B-4064	Italy	LT854057	LT854028	Antonín et al. 2019
89	*Megacollybiaclitocyboidea*	TFB11884/TENN60766	USA	EU623658	–	Hughes et al. 2007
90	*M.clitocyboidea*	TENN62231	USA	EU623664	–	Hughes et al. 2007
91	*M.clitocyboidea*	TENN62230 clone c4	USA	EU623673	–	Hughes et al. 2007
92	*M.clitocyboidea*	TENN62230 clone c5	USA	EU623674	–	Hughes et al. 2007
93	*M.fallax*	MICH 45002	USA	EU623714	–	Hughes et al. 2007
94	*M.fallax*	TFB11561/TENN59447	USA	EU623723	–	Hughes et al. 2007
95	*M.fallax*	DAOM208710	USA	EU623724	–	Hughes et al. 2007
96	*M.fallax*	Mushroom Observer 291302	USA	MN176984	–	Direct Submission
97	*M.fallax*	Mushroom Observer 286893	USA	MT437075	–	Direct Submission
98	*M.marginata*	TENN60752	USA	EU623685	–	Hughes et al. 2007
99	*M.marginata*	HR 91607	Czech	LT854051	–	Antonín et al. 2019
100	*M.platyphylla*	TFB11572/TENN59523	USA	EU623712	–	Hughes et al. 2007
101	*M.platyphylla*	LE 256-2004	USA	EU623713	–	Hughes et al. 2007
102	*M.platyphylla*	10164	Italy	JF908499	–	Osmundson et al. 2013
103	*M.platyphylla*	BRNM 737654	Czech	LT854048	LT854036	Antonín et al. 2019
104	*M.platyphylla*	LE-BIN 3863	Russia	MG734826	–	from GenBank
105	*M.rodmani*	BHS2009-06	USA	GQ397989	–	from GenBank
106	*M.rodmani*	PUL F27039	USA	MW448576	–	from GenBank
107	*M.subfurfuracea*	TFB11075/TENN59558 clone c3	USA	EU623744	–	Hughes et al. 2007
108	*M.subfurfuracea*	TFB11075/TENN59558 clone c8	USA	EU623745	–	Hughes et al. 2007
109	*M.texensis*	DPL7405/TENN62058 clone c1	USA	EU623725	–	Hughes et al. 2007
110	*M.texensis*	DPL7405/TENN62058 clone c2	USA	EU623726	–	Hughes et al. 2007
111	*M.texensis*	FLAS-F-61511	USA	MH211940	–	from GenBank
112	*Mycenapurpureofusca*	HMJAU 43554	China	MG654740	–	Na and Bau 2018
113	*Mycenapurpureofusca*	HMJAU 43624	China	MG654741	–	Na and Bau 2018
114	*Mycenapurpureofusca*	HMJAU 43640	China	MG654742	–	Na and Bau 2018
115	*Porotheleumfimbriatum*	Dai 12276	China	KX081137	KX161656	from GenBank
116	*P.fimbriatum*	Dai 12289	China	KX081138	KX161654	from GenBank
117	*P.fimbriatum*	CLZhao 1120	China	MH114870	–	from GenBank
118	*P.fimbriatum*	CLZhao 2368	China	MH114871	–	from GenBank
119	*P.fimbriatum*	SWFC 006350	China	MK894078	–	from GenBank
120	*P.fimbriatum*	SWFC 006399	China	MK894079	–	from GenBank
121	*Trogiabenghalensis*	CUH AM031	India	KU647630	–	Dutta et al. 2017
122	*T.benghalensis*	CUH AM122	India	MF967246	–	Dutta et al. 2017
123	*T.infundibuliformis*	KUN_HKAS63661	China	JQ031775	JQ031780	Yang et al. 2012
124	*T.infundibuliformis*	KUN_HKAS56709	China	JQ031776	JQ031781	Yang et al. 2012
125	*T.infundibuliformis*	NW1487	Thailand	MW504969	–	Direct Submission
126	*T.venenata*	KUN_HKAS54710	China	JQ031772	JQ031778	Yang et al. 2012
127	*T.venenata*	KUN_HKAS56679	China	JQ031773	JQ031779	Yang et al. 2012
128	*T.venenata*	TC2-28	China	KT968080	–	Mi et al. 2016
129	*T.venenata*	CLZhao 4141	China	MK268886	–	from GenBank

## ﻿Results

### ﻿Phylogenetic analysis

The concatenated dataset of 127 ITS and 50 nLSU sequences from 38 taxa of eight genera in Porotheleaceae, with the addition of one *Mycena* species as an outgroup, comprised 1,527 sites. Sequences retrieved from GenBank and those obtained in this study are listed in Table 1.

**Figure 1.**
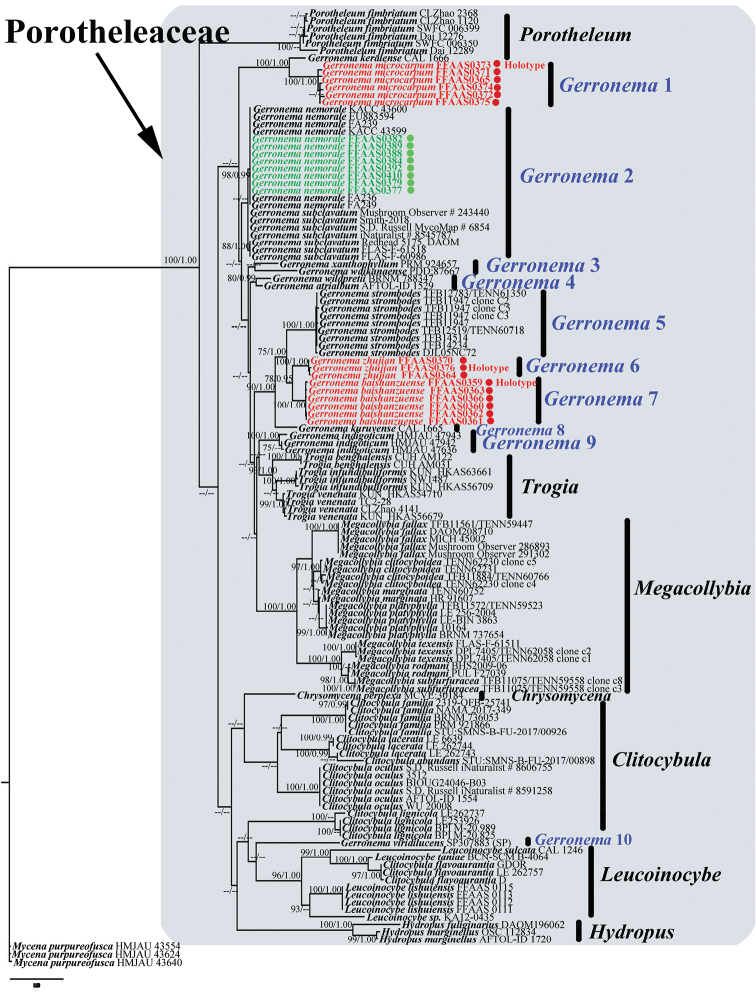
Maximum Likelihood and Bayesian tree concatenated ITS + nLSU dataset. In the generated trees, ML bootstrap support values greater than 75% and Bayesian posterior probabilities (BPP) greater than 0.90 are shown for relevant branch nodes (BS ≥ 75%, BPP ≥ 0.90). The tree is rooted with *Mycenapurpureofusca*. The new species, *Gerronemabaishanzuense*, *G.microcarpum*, and *G.zhujian* are marked by red. The newly discovered species, *G.nemorale* Har. Takah. is marked by green.

BI and ML phylogenetic analyses of the concatenated dataset were performed under the optimal evolutionary model selected for both ITS and nLSU partitions, GTR + I + G (lset nst = 6, rates = gamma, and prset statefreqpr = dirichlet [1,1,1,1]). Because the BI and ML phylogenetic reconstructions were consistent in topology, only the ML tree is shown in Fig. 1.

In the phylogenetic tree shown in Fig. 1, 17 major clades are evident. *Chrysomycena* Vizzini, Picillo, Perrone & Dovana, *Clitocybula*, *Hydropus*, *Leucoinocybe* Singer ex Antonín, Borovička, Holec & Kolařík, *Megacollybia*, *Porotheleum* Fr., and *Trogia* form monophyletic groups, whereas *Gerronema* is polyphyletic (Vizzini et al. 2019). In the analysis of Vizzini et al. (2019), *Gerronema* was resolved into eight clades; in our tree, this number is increased to 10, including 13 species, which we have designated as *Gerronema* clades 1 to 10.

Each individual *Gerronema* clade (e.g., *Gerronema* 1, *Gerronema* 2, etc.) is sister to some subset of Porotheleaceae genera, all with high statistical support (ML bootstrap support [BS] = 100%, Bayesian posterior probability [BPP] = 1.00). Samples of the three new species and the newly recorded species are placed in *Gerronema* 1, *Gerronema* 2, *Gerronema* 6, and *Gerronema* 7 clades, where they constitute monophyletic lineages, each with high statistical support (*G.baishanzuense*, BS = 100%, BPP = 1.00; *G.microcarpum*, BS = 100%, BPP = 1.00; *G.zhujian*, BS = 100%, BPP = 1.00; *G.nemorale*, BS = 98%, BPP = 0.99; Fig. 1). The two new species *G.baishanzuense* and *G.zhujian* form a monophyletic lineage that is sister to a group comprising *Gerronema* 5 and *Gerronema* 8 clades, the latter consisting of *G.strombodes* (Berk. & Mont.) Singer and *G.kuruvense* K.P.D. Latha & Manim. *Gerronemamicrocarpum*, which is well supported as a species, is placed along with *G.keralense* K.P.D. Latha & Manim., a new species recently reported from India, in the *Gerronema* 1 clade (Latha et al. 2018). In contrast, *G.nemorale* is polyphyletic, with accessions of this species and *G.subclavatum* forming an unresolved lineage in the *Gerronema* 2 clade that are difficult to distinguish genetically.

The weakly supported *Gerronema* 3 clade consists of two species: *G.xanthophyllum* (Bres.) Norvell, Redhead & Ammirati and *G.waikanaense* (G. Stev.) J.A. Cooper, collected from the Czech Republic and New Zealand, respectively. Finally, *Gerronema* clades 5 to 10 comprise a single species each.

### ﻿Taxonomy

#### ﻿ Gerronema baishanzuense

Taxon classificationFungiAgaricalesPorotheleaceae

Q. Na, H. Zeng & Y.P. Ge sp. nov.

B8D05290-1A31-5B12-9099-CDAF481C50AD

 842308

Figs 2 , 3 , 4

##### Diagnosis.

Pileus dark brown at center, covered with dark brown fibrillose or pubescent. Stipe densely pruinose when young. Cheilocystidia present. Pileus trama with visible dark brown hyphae and coarse excrescences.

**Figure 2.**
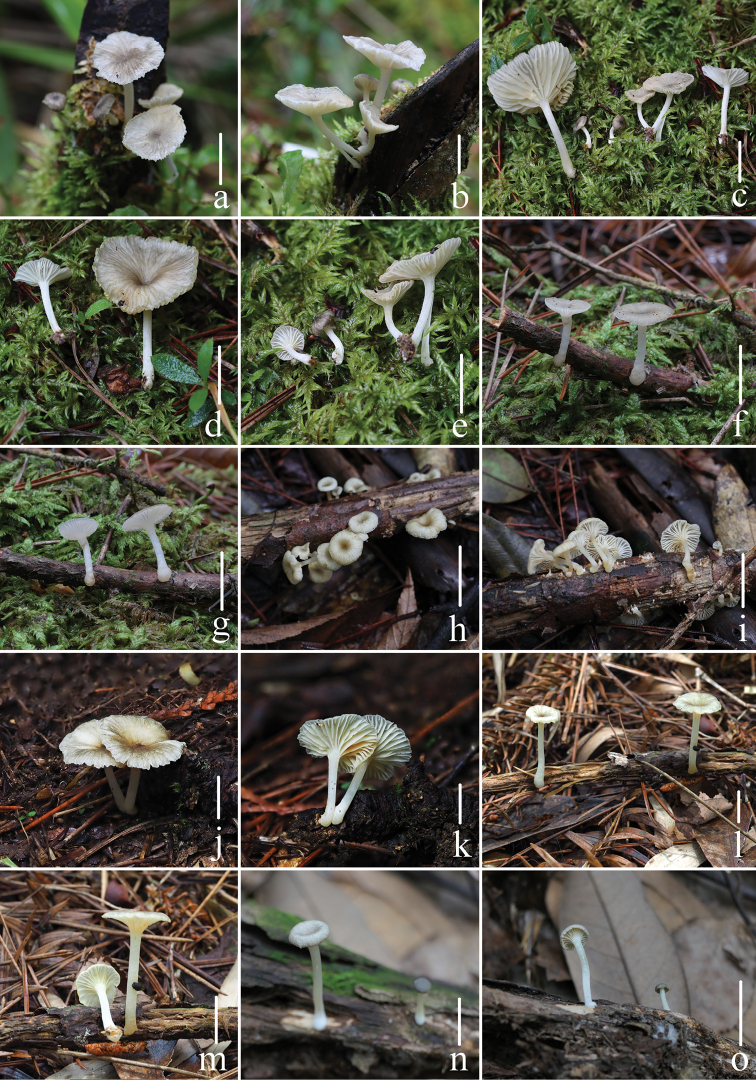
Fresh basidiomata of *Gerronemabaishanzuense* Q. Na, H. Zeng & Y.P. Ge **a–e***FFAAS0359* (Holotype) **f–g***FFAAS0360***h–i***FFAAS0361***j–k***FFAAS0362***l–m***FFAAS0363***n–o***FFAAS0366*. Scale bars: 10 mm (**a–o**). Photographs **a–e** by Qin Na; **f–g** by Junqing Yan **h–i** by Liangliang Qi **j–o** by Yupeng Ge.

##### Holotype.

China. Zhejiang Province, Lishui City, Qingyuan County, Baishanzu, 8 Jul 2020, Qin Na, Yupeng Ge, Yaping Hu, Hui Zeng, and Zewei Liu, *FFAAS0359* (collection no. MY0246).

**Figure 3.**
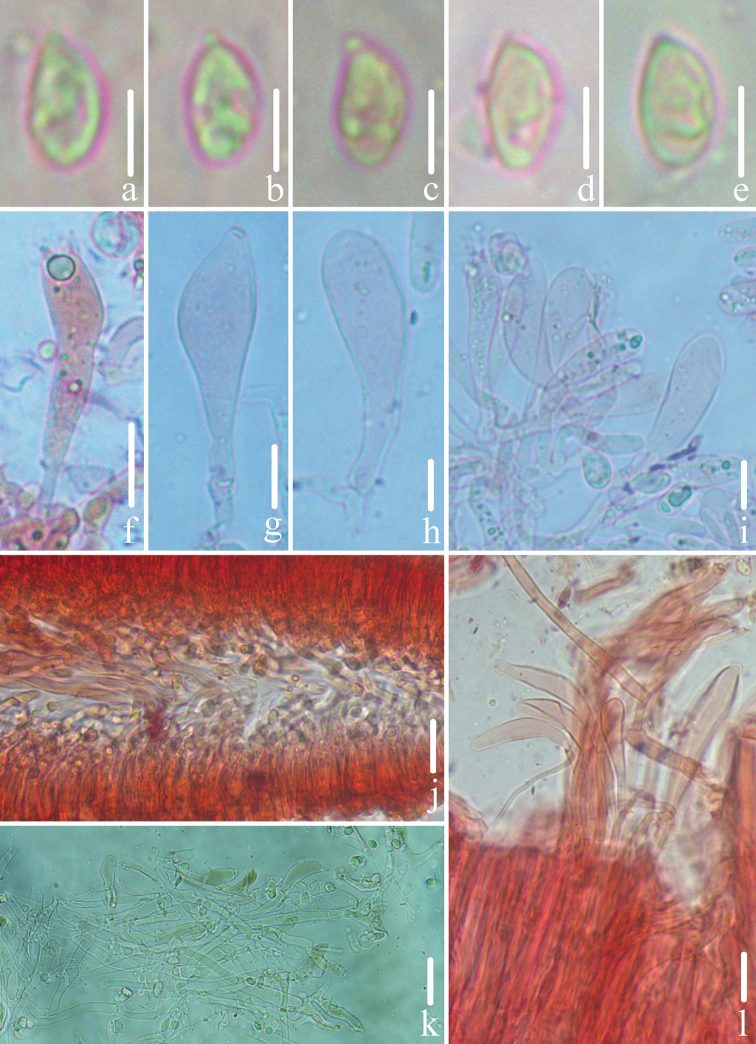
Microscopic features of *Gerronemabaishanzuense* Q. Na, H. Zeng & Y.P. Ge. (*FFAAS0359*, Holotype) **a–e** basidiospores **f** basidia **g–i** cheilocystidia **j** lamellar trama **k** pileipellis **l** stipitipellis and caulocystidia. Scale bars: 5 μm (**a–e**); 10 μm (**f–l**).

##### Etymology.

Refers to the type locality.

##### Description.

Pileus 3.0–25.5 mm in diam., hemispherical when young, becoming applanate and slightly concave at center with age, deeply infundibuliform when old, with uplifted margin, dark brown all over when young (2F8), dark brown at center and fading to light yellowish brown (2D4) towards the margin at maturity, margin light yellowish white (2A2), translucent–striate, sulcate, surface dry, with appressed dark brown (2F8) fibrillose or pubescent, margin glabrescent and brown (2F8), fibrillose or pubescent at the center with age. Context thin and fragile, yellowish white (2A2). Lamellae subdecurrent to decurrent, ascending, cream-white (3A2) to light yellowish white (2A2), faces concolorous with the sides. Stipe slender, 4.5–26.0 × 0.5–2.0 mm, hollow, cylindrical, central, straight, light whitish yellow (4A2), base yellow-brown (4D8) when old, densely pruinose on the entire surface when young, almost glabrous when old, slightly broadened at the base. Odor and taste inconspicuous.

**Figure 4.**
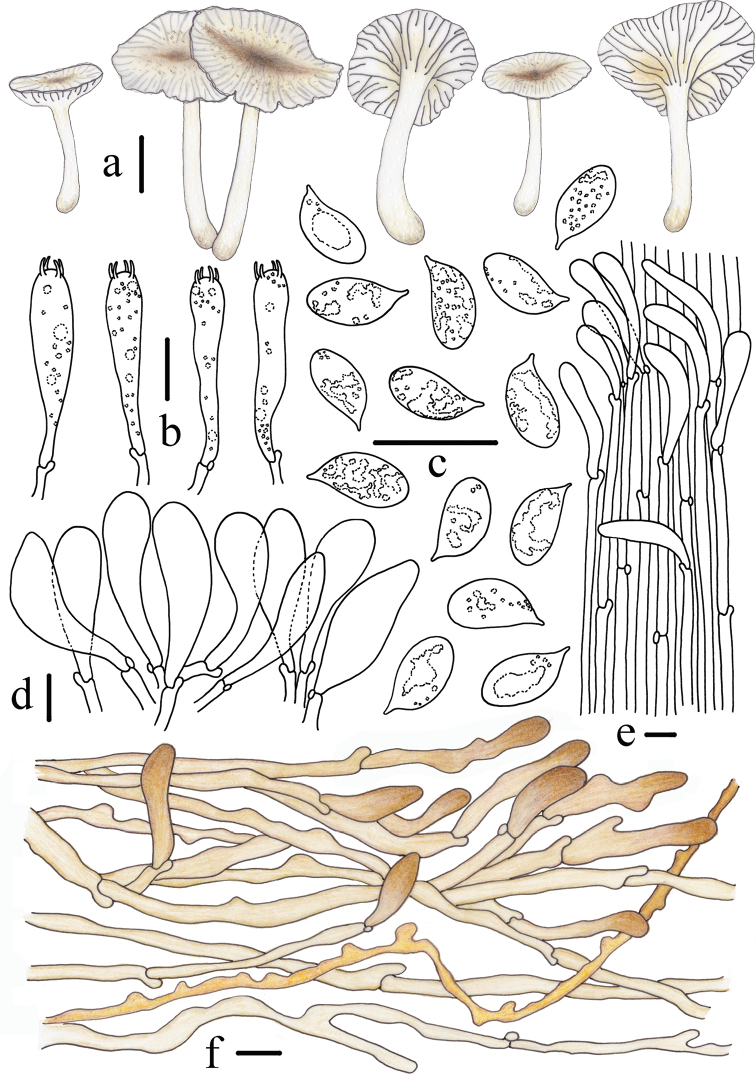
Morphological features of *Gerronemabaishanzuense* Q. Na, H. Zeng & Y.P. Ge. (*FFAAS0359*, Holotype) **a** basidiomata **b** basidia **c** basidiospores **d** cheilocystidia **e** stipitipellis and caulocystidia **f** pileipellis. Scale bars: 10 mm (**a**); 10 μm (**b–f**). Drawing by Qin Na and Yupeng Ge.

Basidiospores [140/7/6] (6.6) 7.5–8.4–9.3 (9.8) × (4.0) 4.4–4.9–5.4 (5.6) μm [*Q* = 1.65–1.74, *Q* = 1.72 ± 0.015] [holotype [40/2/1] (7.6) 7.9–8.6–9.5 (9.8) × (4.3) 4.5–4.9–5.5 (5.6) μm, *Q* = 1.72–1.74, *Q* = 1.74 ± 0.031], long ellipsoid, hyaline, guttulate, thin-walled, inamyloid. Basidia 31–45 × 6–9 μm, hyaline, clavate, 4-spored. Cheilocystidia 30–48 × 8–14 μm, clavate with swollen apex, or subfusiform, hyaline, thin-walled. Pleurocystidia not seen. Lamellar trama subregular; hyphae 2–10 μm wide, thin-walled, hyaline, inamyloid. Pileus trama subregular, sarcodimitic, sometimes with dark brown (4F8) hyphae. Pileipellis a cutis, hyphae 2–6 μm wide, light yellow (2B2) to yellow (2B4), occasionally with coarse excrescences; terminal elements utriform, clavate, sometimes with sparse coarse excrescences, 25–56 × 6–10 μm, light yellowish brown (2C4) to yellowish brown (2C6) pigment in KOH; true pileocystidia absent. Hyphae of the stipitipellis 2–7 μm wide, hyaline, smooth; caulocystidia cylindrical or clavate, 39–70 × 5–14 μm, hyaline, thin-walled. All tissues nonreactive in iodine. Clamps present in all tissues.

##### Habit and habitat.

Solitary to scattered on rotten wood, branches, and twigs in mixed forests of *Picea*, *Pinus*, *Populus*, *Quercus*, etc. Subtropical monsoon climate or subtropical humid climate.

##### Other specimens examined.

Anhui Province, Chizhou City, Shitai County, Dayan Village, Guniujiang National Natural Reserve, 31 Aug 2019, Qin Na, Yupeng Ge, Hui Zeng, Liangliang Qi, and Junqing Yan, *FFAAS0366* (collection no. MY0260); Zhejiang Province, Lishui City, Qingyuan County, Baishanzu, 24 May 2020, Qin Na, Yupeng Ge, Yaping Hu, Hui Zeng, and Zewei Liu, *FFAAS0360* (collection no. MY0247), *FFAAS0362* (collection no. MY0250); Zhejiang Province, Lishui City, Qingyuan County, Jushui Village, 27 May 2020, Qin Na, Yupeng Ge, Yaping Hu, Hui Zeng, and Zewei Liu, *FFAAS0361* (collection no. MY0249), Longquan City, Longquan Mountain, 11 Jul 2020, Qin Na, Yupeng Ge, Yaping Hu, Hui Zeng, and Zewei Liu, *FFAAS0363* (collection no. MY0251).

##### Remarks.

*Gerronemabaishanzuense* is considered to be a distinct species of *Gerronema* on account of its deeply infundibuliform pileus, decurrent lamellae, smooth and long ellipsoid basidiospores, sarcodimitic tramal tissues, cylindrical or clavate caulocystidia, and a lignicolous habitat (Singer 1986; Redhead 1986; Norvell et al. 1994). Four species with a yellow pileus have been recorded: *G.keralense*, *G.kuruvense*, *G.nemorale*, and *G.strombodes* (Singer 1970; Takashi 2009; Antonín et al. 2011; Latha et al. 2018; Takahashi 2000). *Gerronemanemorale*, originally described from Japan and later reported from the Republic of Korea, has the most morphological similarities to *G.baishanzuense*; however, the former differs in having a smaller pileus (< 20 mm in diameter) but a longer stipe (up to 40 mm), terminal elements less than 37 μm long, and much smaller caulocystidia (Takahashi 2000; Antonín et al. 2008). In contrast to *G.baishanzuense*, two new species recently reported from the Indian state of Kerala, *G.keralense* and *G.kuruvense*, are easily mistaken for the new species (Latha et al. 2018). However, the pileus of *G.keralense* lacks dark brown fibrillose or pubescent, has smaller and slightly thick-walled cheilocystidia, and the hyphae of its stipitipellis and caulocystidia are both thin- to thick-walled (Latha et al. 2018). *Gerronemakuruvense* is always distinctly yellow, has small basidiomata (pileus < 11 mm in diameter) and true pileocystidia, and lacks cheilocystidia (Latha et al. 2018). Finally, *G.strombodes*, distributed in North America and Asia, differs from *G.baishanzuense* in having larger basidiomata, a white to grayish white pileus (up to 80 mm wide), smooth pileipellis hyphae, and the absence of hymenial cystidia (Singer 1970; Antonín et al. 2008; Kim et al. 2014). *G.citrinum* (Corner) Pegler (Pegler 1983) and *G.tenue* Dennis (Dennis 1961), are allied with *G.baishanzuense*, but their lamellae edges without cheilocystidia. Moreover, *G.citrinum* has a relatively larger pileus (20–30 mm in diam.) and smaller basidiospores (6–7.5 × 3.5–4 μm), and *G.tenue* differs in having a citrine yellow pileus and an insititious stipe (Dennis 1961; Pegler 1983). *G.hungo* (Henn.) Degreef & Eyi, reported by Degreef and Ndong (2007) as a new combination, differs in yellowish orange to brownish orange pileus, ellipsoid basidiospores, and absent cheilocystidia.

#### ﻿ Gerronema microcarpum

Taxon classificationFungiAgaricalesPorotheleaceae

Q. Na, H. Zeng & Y.P. Ge sp. nov.

E6B18864-0297-53FF-9F4D-F17204446312

 842309

Figs 5 , 6 , 7

##### Diagnosis.

Basidiomata distinctly small. A pileus a bit slimy when moist. Stipe light yellow, base turning to light brown with age. Cheilocystidia common in clavate with rounded apex, rarely fusiform. Pileipellis occasionally with coarse excrescences.

**Figure 5.**
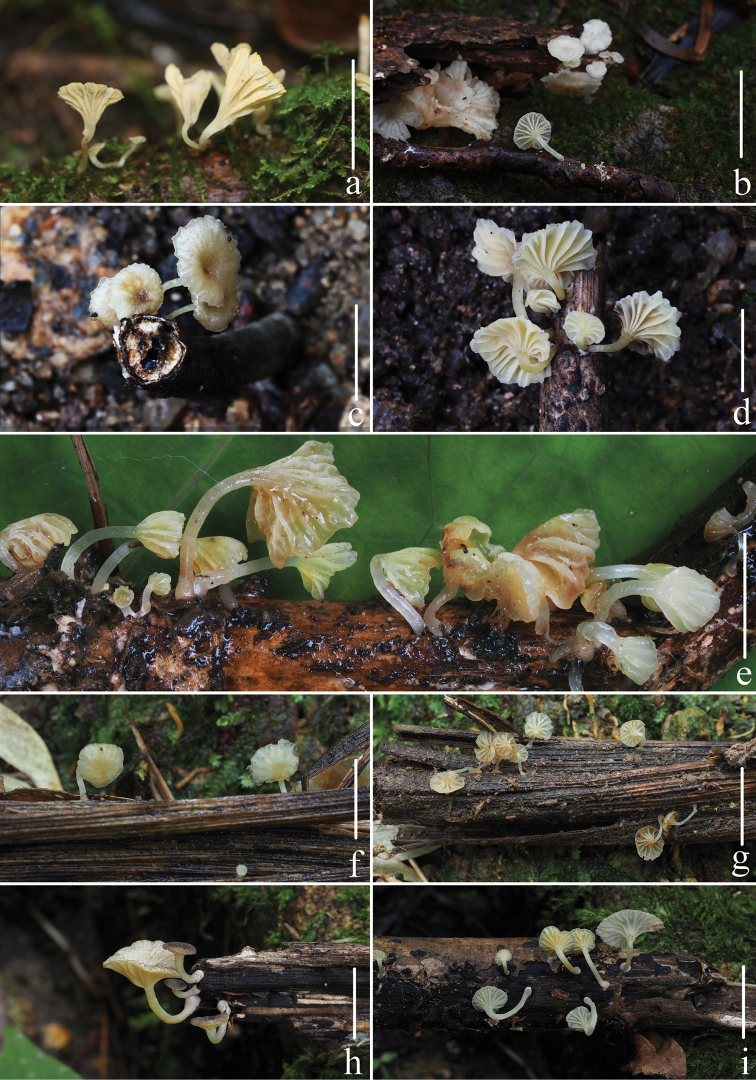
Fresh basidiomata of *Gerronemamicrocarpum* Q. Na, H. Zeng & Y.P. Ge. **a***FFAAS0365***b***FFAAS0372***c–d***FFAAS0375***e***FFAAS0373* (Holotype) **f–g***FFAAS0374***h–i***FFAAS0371*. Scale bars: 10 mm (**a–i**). Photographs **a, e–i** by Yupeng Ge; **b** by Junqing Yan; **c–d** by Qin Na.

##### Holotype.

China. Zhejiang Province, Lishui City, Qingtian County, Shigu Lake, 6 Aug 2021, Qin Na, Yupeng Ge, Junqing Yan, Zewei Liu, and Yulan Sun, *FFAAS0373* (collection no. MY0526).

**Figure 6.**
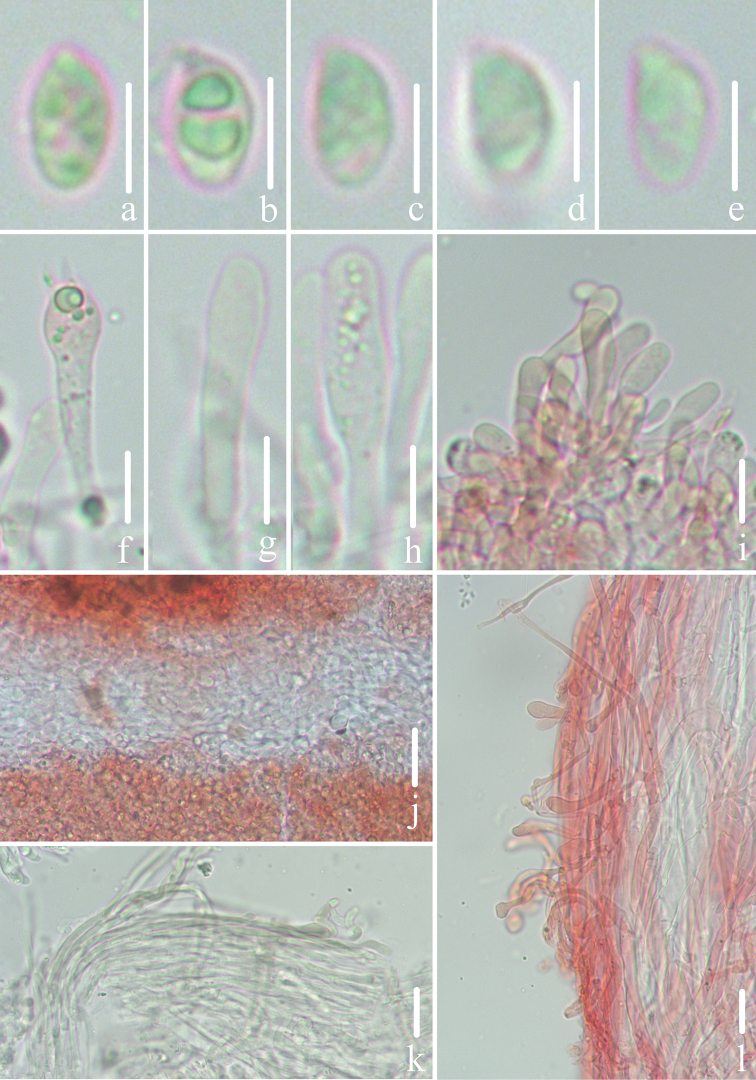
Microscopic features of *Gerronemamicrocarpum* Q. Na, H. Zeng & Y.P. Ge. (*FFAAS0373*, Holotype) **a–e** basidiospores **f** basidia **g–i** cheilocystidia **j** lamellar trama **k** pileipellis **l** stipitipellis and caulocystidia. Scale bars: 5 μm (**a–e**); 10 μm (**f–l**).

##### Etymology.

Refers to the small basidiomata.

##### Description.

Pileus 1.5–9.0 mm in diam., at first convex, later applanate in the marginal zone, infundibuliform or deeply umbilicate in the center when old, grayish yellow (2B2) to shallow yellowish brown (2C4), shallowly sulcate, translucent–striate, smooth, a bit slimy when moist, but not hygrophanous. Context yellowish white (2A2), thin. Lamellae close to moderately close, shortly decurrent when young, whitish yellow (1A2), decurrent to deeply decurrent when old, concolorous with the sides. Stipe 5.0–18.0 × 1.0–2.0 mm, hollow or soon becoming hollow, generally central, equal or with slightly broader base, light yellow (2A2), becoming light brown (5C6) towards the base, pruinose, glabrescent when old, base covered with a few white fibrils. Odor and taste indistinctive.

**Figure 7.**
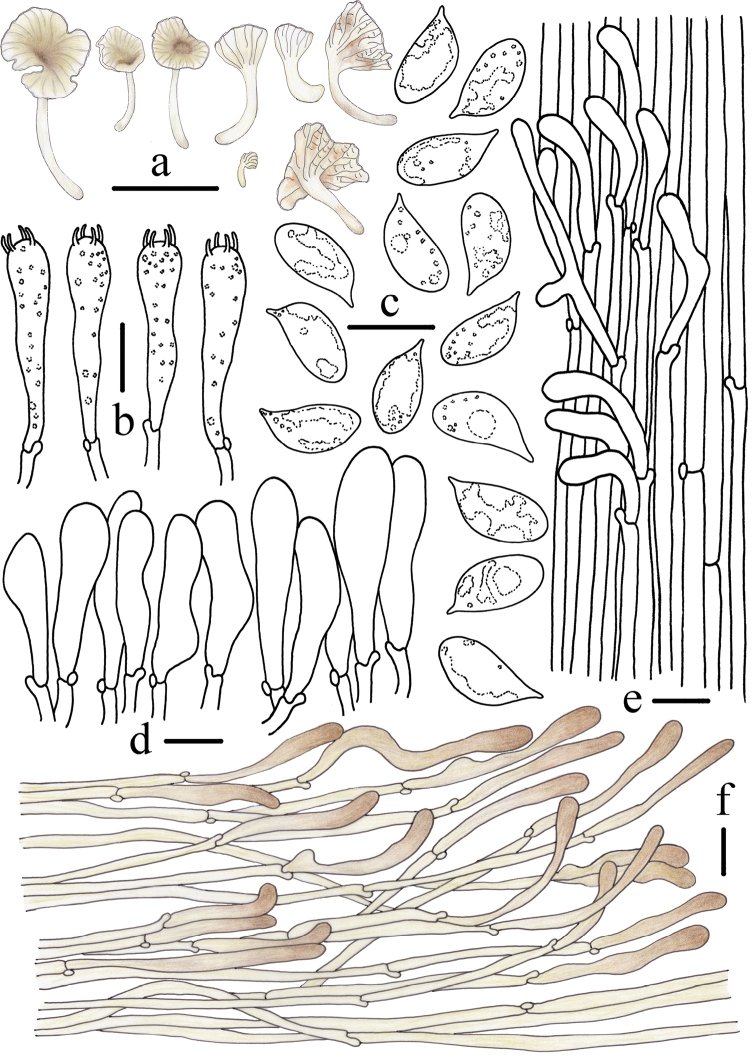
Morphological features of *Gerronemamicrocarpum* Q. Na, H. Zeng & Y.P. Ge. (*FFAAS0373*, Holotype) **a** basidiomata **b** basidia **c** basidiospores **d** cheilocystidia **e** stipitipellis and caulocystidia **f** pileipellis. Scale bars: 10 mm (**a**); 10 μm (**b, d–f**); 5 μm (**c**). Drawing by Qin Na and Yupeng Ge.

Basidiospores [140/7/6] (6.1) 6.3–6.8–7.2 (7.5) × (3.3) 3.5–3.8–4.1 (4.3) μm [*Q* = 1.64–1.95, *Q* = 1.80 ± 0.059] [holotype [40/2/1] (6.1) 6.2–6.7–7.3 (7.5) × 3.4–3.7–4.1 (4.3) μm, *Q* = 1.64–1.95, *Q* = 1.81 ± 0.066], narrowly ellipsoid to cylindrical, hyaline in water and 5% KOH, inamyloid, smooth. Basidia 25–33 × 6–8 μm, 4-spored, clavate, hyaline. Cheilocystidia common in clavate with rounded apex, 31–35 × 5–8 μm, rarely fusiform, thin-walled and hyaline. Pleurocystidia not seen. Lamellar trama subregular; hyphae 2–5 μm wide, thin-walled, hyaline, inamyloid. Pileus trama subregular, sarcodimitic. Pileipellis a cutis, hyphae 3–6 μm wide, light yellow (2B2); terminal elements clavate, utriform, occasionally with coarse excrescences, 19–43 × 4–6 μm, light yellowish brown (2C4) to yellowish brown (2D4) pigment in KOH; true pileocystidia absent. Hyphae of the stipitipellis 2–6 μm wide, hyaline, smooth; caulocystidia long cylindrical or clavate, 26–65 × 4–9 μm, hyaline, thin-walled. All tissues nonreactive in iodine. Clamps present in all tissues.

##### Habit and habitat.

Scattered on rotten wood and twigs in mixed evergreenbroadleaf forests consisting of species of Fagaceae, Lauraceae, Theaceae, Ericaceae, Symplocaceae, Pinaceae, etc. Subtropical monsoon climate or subtropical humid climate.

##### Other specimens examined.

Anhui Province, Chizhou City, Shitai County, Dayan Village, Guniujiang National Natural Reserve, 31 Aug 2019, Qin Na, Yupeng Ge, Hui Zeng, Liangliang Qi, and Junqing Yan, *FFAAS0365* (collection no. MY0259); Fujian Province, Nanping City, Wuyi Mountain, 25 Jul 2020, Qin Na, Yupeng Ge, Yaping Hu, Hui Zeng, and Zewei Liu, *FFAAS0375* (collection no. MY0544); Zhejiang Province, Hangzhou City, Tianmu Mountain, 30 Jul 2021, Qin Na, Yupeng Ge, Zewei Liu, and Yulan Sun, *FFAAS0371* (collection no. MY0424); Lishui City, Liandu District, Baiyun National Forest Park, 2 Aug 2021, Qin Na, Yupeng Ge, Zewei Liu, and Yulan Sun, *FFAAS0372* (collection no. MY0478), Qingtian County, Shigu Lake, 6 Aug 2021, Qin Na, Yupeng Ge, Junqing Yan, Zewei Liu, and Yulan Sun, *FFAAS0374* (collection no. MY0527).

##### Remarks.

Characteristics such as tiny omphalinoid basidiomata, decurrent lamellae, inamyloid and narrowly ellipsoid to cylindrical basidiospores, sarcodimitic tramal tissues, a pileipellis with pigmented terminal elements, and long cylindrical or clavate caulocystidia support the placement of this species in *Gerronema* (Singer 1970, 1986; Norvell et al. 1994). Because of its small basidiomata, decurrent lamellae, and subregular pileus trama, *G.kuruvense* is difficult to distinguish from *G.microcarpum*, but its pileus is orange yellow all over, no cheilocystidia or pleurocystidia are present, and its pileocystidia and caulocystidia are somewhat thick-walled (Latha et al. 2018). *Gerronemanemorale* has certain morphological similarities to *G.microcarpum*, namely, the presence of tiny yellowish basidiomata, decurrent lamellae, and cylindrical basidiospores (Antonín et al. 2008, 2011; Takashi 2009). However, *G.nemorale* differs in having a pileus with an olive tint, a longer stipe with conspicuous white mycelioid bristles, and larger terminal elements of the pileipellis (up to 150 μm) (Antonín et al. 2008, 2011; Takashi 2009). Compared with *G.microcarpum*, *G.subchrysophyllum* (Murrill) Singer has an olive-umber pileus fading to grayish when old, larger and ellipsoid basidiospores (4.3–8.5 × 2.5–6.3 μm), and sometimes basidiole-like cheilocystidia (Singer 1970). *Gerronemakeralense* and *G.strombodes* are easily mistaken for *G.microcarpum*, but both the two closely related species are distinguishable by their absence of cheilocystidia or their partially thick-walled pileipellis and stipitipellis (Singer 1970; Antonín et al. 2008; Latha et al. 2018; Kim et al. 2014).

#### ﻿ Gerronema zhujian

Taxon classificationFungiAgaricalesPorotheleaceae

Q. Na, H. Zeng & Y.P. Ge sp. nov.

ED149D7D-F569-5632-A8FF-4B06FCAC6433

 842310

Figs 8 , 9 , 10

##### Diagnosis.

Pileus fuscous and densely covered with tiny, deep brown fur or scales, distinctly radially striped with darkened lines. Stipe white, upper part slight brown when old. Cheilocystidia present. Pileipellis without coarse excrescences.

**Figure 8.**
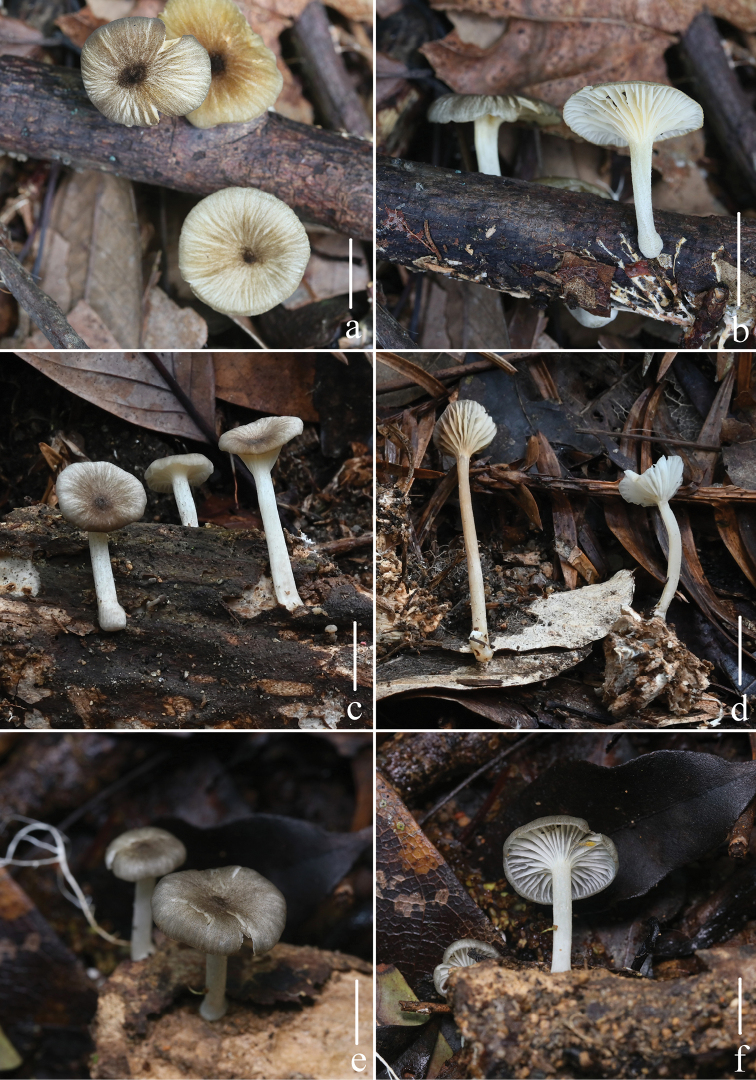
Fresh basidiomata of *Gerronemazhujian* Q. Na, H. Zeng & Y.P. Ge. **a–b***FFAAS0364***c–d***FFAAS0376* (Holotype) **e–f***FFAAS0370*. Scale bars: 10 mm (**a–f**). Photographs **a–b, e–f** by Liangliang Qi **c–d** by Junqing Yan.

##### Holotype.

China. Fujian Province, Nanping City, Wuyi Mountain, 25 Jul. 2020, Qin Na, Yupeng Ge, Yaping Hu, Hui Zeng, and Zewei Liu, *FFAAS0376* (collection no. MY0553).

**Figure 9.**
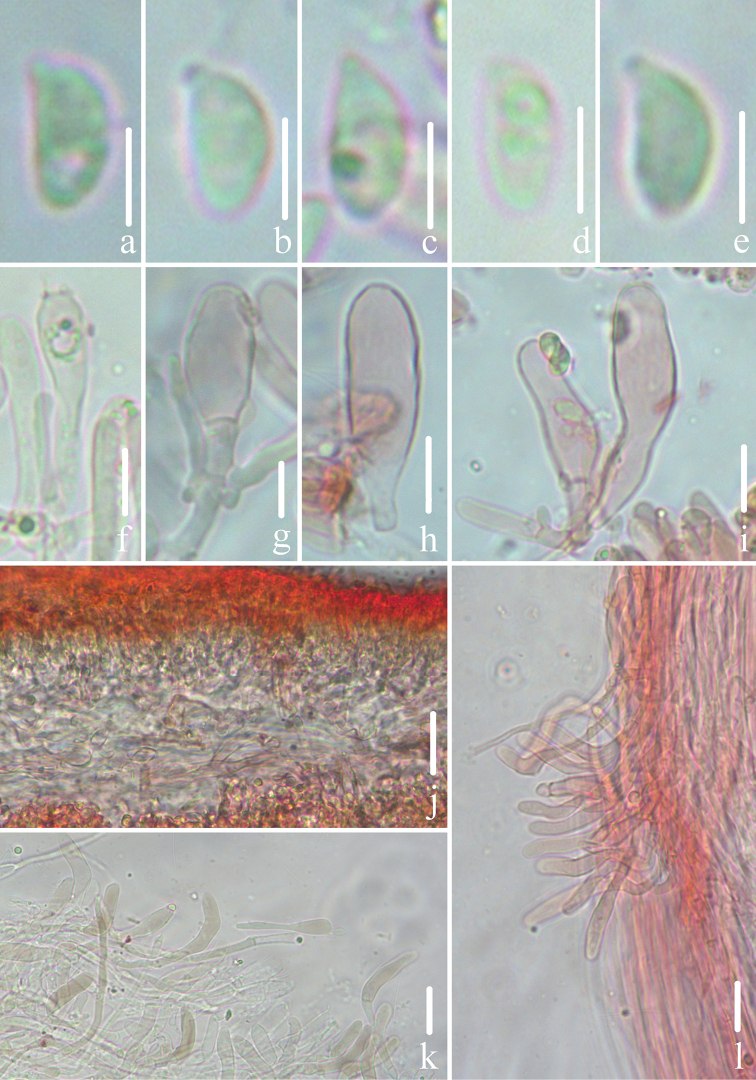
Microscopic features of *Gerronemazhujian* Q. Na, H. Zeng & Y.P. Ge. (*FFAAS0376*, Holotype) **a–e** basidiospores **f** basidia **g–i** cheilocystidia **j** lamellar trama **k** pileipellis **l** stipitipellis and caulocystidia. Scale bars: 5 μm (**a–e**); 10 μm (**f–l**).

##### Etymology.

The name refers to the centrally depressed, umbilicate basidiocarps, which resemble an eye or a loudspeaker; zhujian is a mythical one-eyed Chinese creature who is usually very noisy, like a walking loudspeaker.

**Figure 10.**
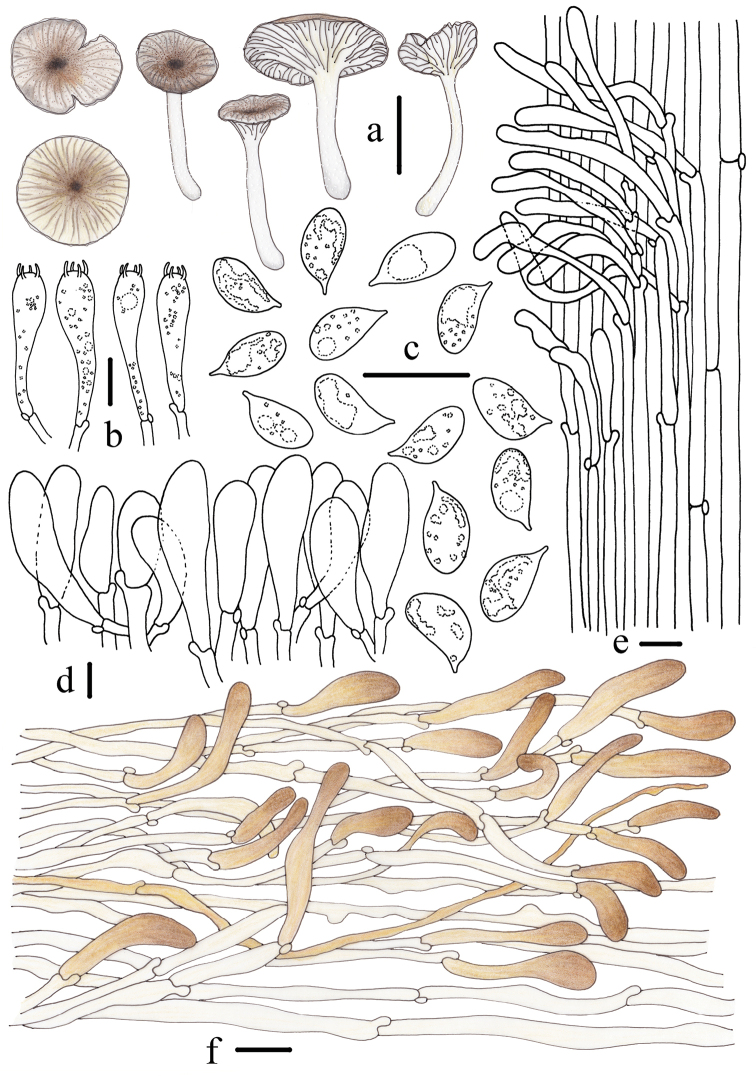
Morphological features of *Gerronemazhujian* Q. Na, H. Zeng & Y.P. Ge. (*FFAAS0376*, Holotype) **a** basidiomata **b** basidia **c** basidiospores **d** cheilocystidia **e** stipitipellis and caulocystidia **f** pileipellis. Scale bars: 10 mm (**a**); 10 μm (**b–f**). Drawing by Qin Na and Yupeng Ge.

##### Description.

Pileus 8.6–18.5 mm in diam., convex to broadly convex, papillate, applanate and centrally depressed, subumbilicate to umbilicate with age, pellucid-striate to rugulo-striate, or sulcate, always ± distinctly radially striped with darkened lines, fuliginous-fuscous (2F8) or fuscous (4F8) at center when young, grayish white (3B1) towards the margin, fading to brown (3F8) at the center, yellowish-brown (4E8) towards the margin, densely covered with tiny, deep brown (4F4) fur or scales, slightly sparse with age, with a slightly involuted margin. Context white, thin, tough. Lamellae subdecurrent to decurrent, moderately broad, pure white to yellowish-white (4A2), edges concolorous with the sides. Stipe 19.0–25.0 × 1.0–1.5 mm, central, cylindrical, almost equal above, white, slight brown (8D3–8D4) in upper part when old, fibrous, hollow, pruinose, base slightly swollen with tiny, white fine hairs. Odorless, taste mild.

Basidiospores [80/4/3] (6.3) 6.7–7.4–8.0 (8.5) × (3.2) 3.7–4.1–4.6 (4.8) μm [*Q* = 1.64–2.07, *Q* = 1.81 ± 0.076] [holotype [40/2/1] (6.3) 6.6–7.4–7.9 (8.3) × (3.2) 3.7–4.0–4.5 (4.6) μm, *Q* = 1.69–2.07, *Q* = 1.82 ± 0.087], narrowly ellipsoid to cylindrical, hyaline, guttulate, thin-walled, inamyloid. Basidia 28–40 × 6–9 μm, hyaline, clavate, 4-spored. Cheilocystidia 29–46 × 7–13 μm, subfusiform, clavate, apex usually swollen, hyaline. Pleurocystidia absent. Lamellar trama subregular; hyphae 3–8 μm wide, thin-walled, hyaline, inamyloid. Pileus trama subregular, sarcodimitic. Pileipellis hyphae 3–6 μm wide, a cutis, light yellow (2B2); terminal elements utriform or clavate, 25–49 × 6–9 μm, light yellowish brown (2C4) to yellowish brown (2C6) pigmented, especially in the apex; true pileocystidia absent. Hyphae of the stipitipellis 2–8 μm wide, hyaline, smooth; caulocystidia long cylindrical, sometimes with rounded apex, 27–47 × 4–8 μm, hyaline, thin-walled. All tissues nonreactive in iodine. Clamps present in all tissues.

##### Habit and habitat.

Solitary to scattered on rotten wood, branches, and twigs in Theaceae, Fagaceae, Symplocaceae, Lauraceae, Aquifoliaceae, Ericaceae, and Pinaceae mixed forests. Subtropical monsoon climate, subtropical humid climate or subtropical maritime monsoon climate.

##### Other specimens examined.

Anhui Province, Chizhou City, Shitai County, Dayan Village, Guniujiang National Natural Reserve, 26 Jul 2019, Qin Na, Yupeng Ge, Hui Zeng, Junqing Yan, and Liangliang Qi, *FFAAS0364* (collection no. MY0256); Fujian Province, Sanming City, Mingxi County, Junzifeng National Natural Reserve, 23 Jun 2021, Qin Na, Yupeng Ge, Liangliang Qi, and Binrong Ke, *FFAAS0370* (collection no. MY0296).

##### Remarks.

*Gerronemazhujian* is unique among *Gerronema* on account of its fuscous pileus with tiny, dark brown fur or scales, its distinctly radially striping with darkened lines, cheilocystidia present and pileipellis without coarse excrescences. Two species of *Omphalina* characterized by dark pigments in the pileus–*Omphalinadepauperata* (Singer) Raithelh. and *O.subpallida* (Singer) Raithelh., formerly named *G.subpallidum* Singer and *G.depauperatum* Singer, respectively, have been described from Argentina. These two species most closely resemble *G.zhujian* but differ in having a hyaline or white stipe, ellipsoid basidiospores, and no cheilocystidia (Singer 1970). *Gerronemachrysocarpum* is closely allied to *G.zhujian* on the basis of the dark brown coloration of the umbilicus of its pileus, its whitish stipe, and similarly shaped basidiospores (Liu 1995). This taxon differs from *G.zhujian* in having a viscid and glabrescent pileus, pale orange lamellar margin, and pleurocystidia (Liu 1995). Other species of *Gerronema*, such as *G.nemorale* and *G.strombodes*, have a distinctly yellow, yellowish orange, olive yellow to yellowish brown pileus, and their micromorphological features are also different (Singer 1970; Antonín et al. 2008; Latha et al. 2018).

#### ﻿ Gerronema nemorale

Taxon classificationFungiAgaricalesPorotheleaceae

Har. Takah.

CEDD83BD-A606-50EB-AE09-328F3FFE7626

Figs 11 , 12 , 13

##### Description.

Pileus 3.0–19.0 mm in diam., hemispherical at first, then convex with a depressed center, applanated and deeply umbilicate with age, slightly striate at the margin in younger basidiomata, slightly translucently striate forming shallow grooves, greenish yellow (2E3), yellowish brown (2D5), olive brown (2E8), always deeper at the center, fading light yellow (5A2) towards the margin, finely tomentose when young, glabrescent with age, with a flat margin. Context white to light yellow, thin. Lamellae moderately distant to distant, decurrent, white or pale yellow (5A2), narrow, edges concolorous with the sides. Stipe 19.0–36.0 × 1.0–2.5 mm, almost equal, but swollen at the base, terete, slender, hollow, pruinose overall, glabrescent with age; base with conspicuous white mycelioid bristles. Odorless, taste mild.

**Figure 11.**
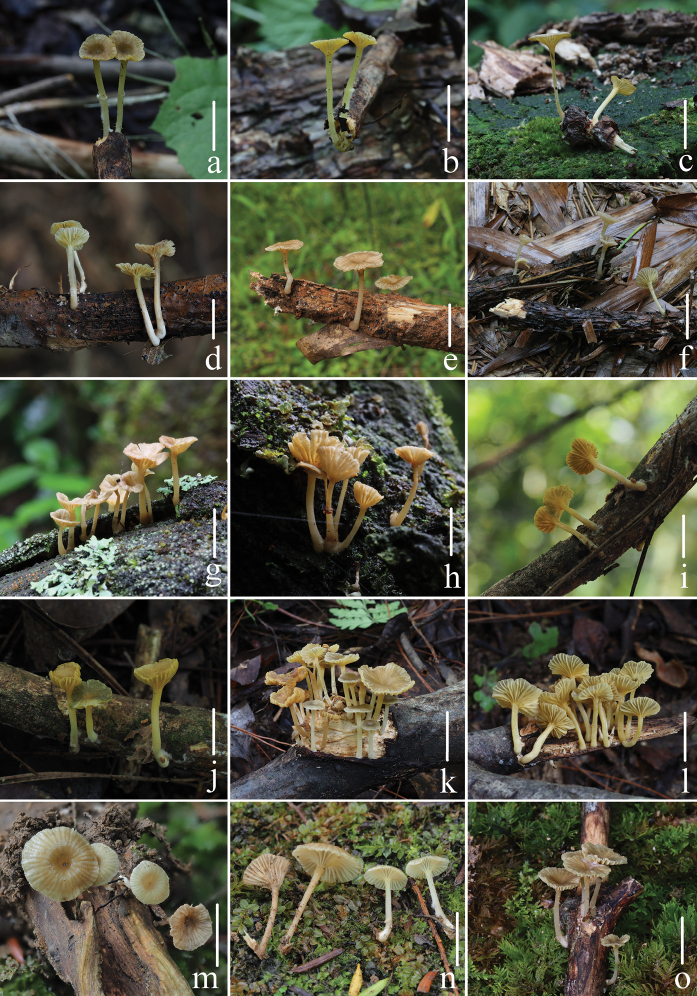
Fresh basidiomata of *Gerronemanemorale* Har. Takah. **a–b***MY0364* (Wunvfeng, Jian City, Liaoning Province) **c***MY0113* (Guniujiang, Shitai County, Anhui Province) **d***MY0264* (Miaoqian Town, Qingyang County, Anhui Province) **e***MY0248* (Baishanzu, Qingyuan County, Zhejiang Province) **f***MY0254* (Longquan Mountain, Longquan City, Zhejiang Province) **g–h***MY0273* (Lanni Lake, Qingtian County, Zhejiang Province) **i–j***MY0276* (Dayang Mountain, Jinyun County, Zhejiang Province) **k–l***MY0462* (Baiyun Forest Park, Lishui City, Zhejiang Province) **m–n***MY0287* (Junzifeng, Sanming City, Fujian Province) **o***MY0549* (Wuyi Mountain, Nanping City, Fujian Province). Scale bars: 10 mm (**a–o**). Photographs **a–e** by Qin Na; **f–g** by Junqing Yan; **h–i** by Liangliang Qi; **j–o** by Yupeng Ge.

Basidiospores [60/3/3] (6.8) 7.9–8.8–9.9 (10.7) × (3.7) 4.6–5.2–5.8 (6.3) μm [*Q* = 1.59–1.88, *Q* = 1.70 ± 0.065], narrowly ellipsoid or cylindrical, hyaline, guttulate, thin-walled, inamyloid. Basidia 32–46 × 6–9 μm, hyaline, clavate, 4-spored. Cheilocystidia 27–49 × 5–9 μm, abundant, irregularly cylindric or clavate, colorless. Pleurocystidia absent. Lamellar trama subregular; hyphae 3–9 μm wide, thin-walled, hyaline, inamyloid. Pileus trama subregular, sarcodimitic. Pileipellis hyphae 2–5 μm wide, light yellow (2B2), a cutis; terminal elements cylindric or clavate, 31–50 × 4–9 μm, light yellowish brown (2C4) to yellowish brown (2C6) pigmented, especially in the apex; true pileocystidia absent. Hyphae of the stipitipellis 3–6 μm wide, hyaline, smooth; caulocystidia cylindrical or broadly clavate, 32–48 × 5–8 μm, hyaline, thin-walled. All tissues nonreactive in iodine. Clamps present in all tissues.

**Figure 12.**
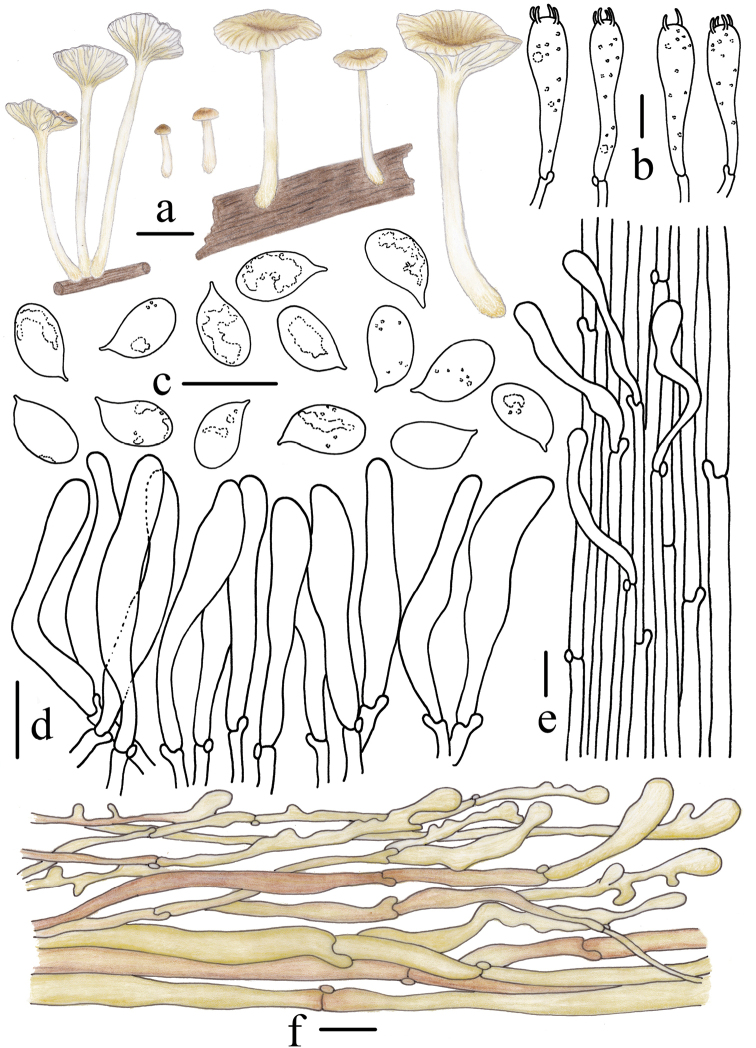
Morphological features of *Gerronemanemorale* Har. Takah. (*FFAAS0410*) **a** basidiomata **b** basidia **c** basidiospores **d** cheilocystidia **e** stipitipellis and caulocystidia **f** pileipellis. Scale bars: 10 mm (**a**); 10 μm (**b–f**). Drawing by Qin Na and Yupeng Ge.

##### Habit and habitat.

Solitary to caespitose on dead fallen twigs or rotten wood in mixed broadleaf–conifer forests from early spring to late autumn, common, especially in subtropical zones in China. Subtropical monsoon climate, subtropical humid climate subtropical maritime monsoon climate, or continental monsoon humid climate.

**Figure 13.**
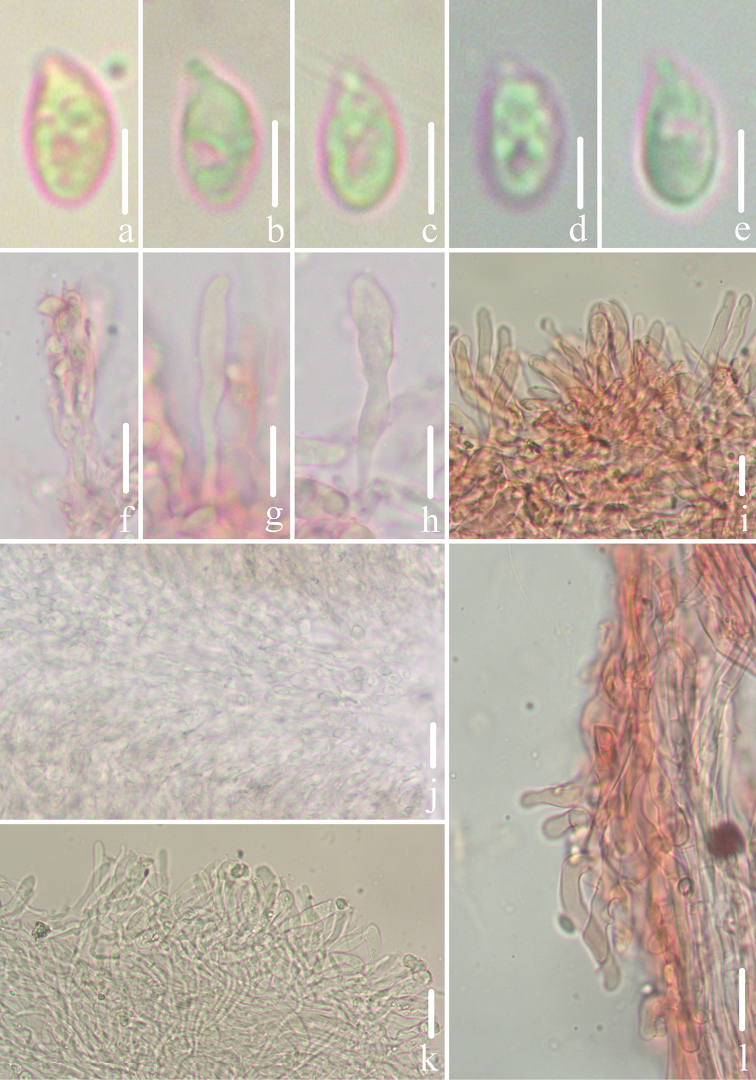
Microscopic features of *Gerronemanemorale* Har. Takah. (*FFAAS0410*) **a–e** basidiospores **f** basidia **g–i** cheilocystidia **j** lamellar trama **k** pileipellis **l** stipitipellis and caulocystidia. Scale bars: 5 μm (**a–e**); 10 μm (**f–l**).

##### Distribution.

Known from Asia (Japan, Korea, Pakistan).

##### Specimens examined.

Anhui Province, Chizhou City, Shitai County, Dayan Village, Guniujiang National Natural Reserve, 7 Jun 2019, Qin Na, Yupeng Ge, Hui Zeng, Junqing Yan, and Liangliang Qi, *FFAAS0377* (collection no. MY0113), Qingyang County, Miaoqian Town, 2 Sep 2019, Qin Na, Yupeng Ge, Hui Zeng, Junqing Yan, and Liangliang Qi, *FFAAS0384* (collection no. MY0264); Fujian Province, Nanping City, Wuyi Mountain, 10 Aug 2021, Qin Na, Yupeng Ge, Junqing Yan, Zewei Liu, and Yulan Sun, *FFAAS0410* (collection no. MY0549), Sanming City, Junzifeng National Natural Reserve, 22 Jun 2021, Qin Na, Yupeng Ge, Binrong Ke, and Liangliang Qi, *FFAAS0390* (collection no. MY0287); Zhejiang Province, Lishui City, Qingyuan County, Wangmu, 26 May 2020, Qin Na, Yupeng Ge, Yaping Hu, Junqing Yan, and Zewei Liu, *FFAAS0379* (collection no. MY0248); Jilin Province, Tonghua City, Jian City, Wunvfeng National Forest Park, 6 Jul 2021, Qin Na, Yupeng Ge, and Zewei Liu, *FFAAS0392* (collection no. MY0364); Zhejiang Province, Lishui City, Longquan City, Zhuangbian Village, 10 Jul 2020, Qin Na, Yupeng Ge, Junqing Yan, and Zewei Liu, *FFAAS0382* (collection no. MY0254), Liandu District, Baiyun National Forest Park, 2 Aug 2021, Qin Na, Zewei Liu, *FFAAS0395* (collection no. MY0462), Qingtian County, Lanni Lake, 2 Jun 2021, Qin Na, Yupeng Ge, Junqing Yan, Yulan Sun, and Zewei Liu, *FFAAS0388* (collection no. MY0273), Jinyun County, Dayang Mountain, 3 Jun 2021, Qin Na, Yupeng Ge, and Junqing Yan, *FFAAS0389* (collection no. MY0276).

##### Remarks.

Having a sarcodimitic tissue structure, *G.nemorale* fits well within the currently restricted concept of the genus *Gerronema* (Redhead 1986; Norvell et al. 1994). *Gerronemanemorale* seems to be rather common in the East Asian region (Takahashi 2000; Antonín et al. 2008; Kim et al. 2014; Aqdus and Khalid 2021). Kim et al. (2014) has reported a basidiospore size of 6.0–8.2 × 3.5–4.8 μm for *G.nemorale* collected from Mount Halla (Jeju Island) in southwestern Korea, which is distinctly smaller than that of other specimens from Korea, Japan, Pakistan, and our collections (Takahashi 2000; Antonín et al. 2008; Kim et al. 2014; Aqdus and Khalid 2021). *Gerronematenue* Dennis, described from Venezuela, is allied with *G.nemorale*, but the latter differs in having a citrine yellow pileus, an insititious stipe, and lamellae edges without cheilocystidia (Dennis 1961). Another similar species, *G.corticiphilum* Lj.N. Vassiljeva, described as *G.corticiphila*, has a rarely sulfurous-colored pileus and larger and narrower basidiospores (13–17 × 4–5 μm) (Vassiljeva 1973). In addition, *G.icterinum* (Singer) Singer from South America, now treated as *Trogiaicterina* (Singer) Corner, shows some similarities with *G.nemorale* but has veined and forked lamellae and lacks cheilocystidia (Singer 1986). Another species of *Trogia*, *T.mellea* Corner, is also similar to *G.nemorale*, but can be easily distinguished from the latter in having no olivaceous tones on the pileus surface, a fuscous, pruinose pileus center and stipe, and subclavate or subventricose cheilocystidia (Corner 1966).

## ﻿Discussion

Our phylogenetic analysis divided *Gerronema* into several highly supported clades containing other members of Porotheleaceae, thus providing further evidence that *Gerronema* is polyphyletic (Vizzini et al. 2019). This finding is consistent with the view of Vizzini et al., who only included seven genera in Porotheleaceae: *Hydropus*, *Chrysomycena*, *Clitocybula*, *Leucoinocybe*, *Megacollybia*, *Porotheleum*, and *Trogia*; in addition, many taxa in this family in the sense of Redhead have a sarcodimitic structure (Redhead 1986; Vizzini et al. 2019). The phylogenetic results are in agreement with the taxonomic concept of *Gerronema* as a heterogeneous group. Although *Gerronema* was treated after its establishment as a subgenus of *Omphalina* by Lange (1981), the view of *Gerronema* as a distinct genus has been widely adopted (Redhead 1986; Norvell et al. 1994).

The phylogenetically and morphologically closest genera to *Gerronema* are *Megacollybia* and *Trogia* (Hughes et al. 2007; Antonín et al. 2019; Vizzini et al. 2019). Compared with *Gerronema*, however, *Megacollybia* is well characterized by the presence of rhizomorphs at the base of stipe and a sarcodimitic stipe structure, whereas narrow and frequently forked gills and a trichodermic pileipellis are observed in *Trogia* (Corner 1966; Hughes et al. 2007). Other groups in the same family, namely, *Hydropus*, *Chrysomycena*, *Clitocybula*, *Leucoinocybe*, and *Porotheleum*, have different morphological characteristics and are genetically distant from *Gerronema* (Hausknecht et al. 1997; Antonín et al. 2008, 2019; Vizzini et al. 2019).

Since 1995, only three species of *Gerronema* have been reported from China, namely, *G.albidum* (Fr.) Singer, *G.chrysocarpum* P.G. Liu, and *G.indigoticum* T. Bau & L.N. Liu (Liu 1995; Dai et al. 2010; Liu et al. 2019). The distinctly white and blue basidiomata of *G.albidum* and *G.indigoticum* can be used to distinguish those two species from our newly described and newly recorded species, and *G.chrysocarpum* has a viscid pileus and pleurocystidia (Liu 1995; Dai et al. 2010; Liu et al. 2019).

### ﻿Key to seven species of *Gerronema* in China

**Table:** 

1	Basidiomata not yellow or brown	**2**
–	Basidiomata yellow to brown	**3**
2	Pileus and stipe blue	***G.indigoticum***
–	Pileus and stipe white	***G.albidum***
3	Pleurocystidia present	***G.chrysocarpum***
–	Pleurocystidia absent	**4**
4	Pileus densely covered with deep brown fur or scales	***G.zhujian***
–	Pileus without fur or scales	**5**
5	Basidiomata distinctly small (Pileus < 9 mm in diam.)	***G.microcarpum***
–	Basidiomata moderately small (Pileus > 9 mm in diam.)	**6**
6	Cheilocystidia up to 48 μm	***G.baishanzuense***
–	Cheilocystidia less than 35 μm	***G.nemorale***

Morphological and molecular evidence support classification of the four newly recognized/recorded species as members of *Gerronema*. The four species share an umbonate or infundibuliform pileus, decurrent lamellae, inamyloid basidiospores, clavate cystidia, and sarcodimitic tramal tissues. In addition, the four species are lignicolous in habit, growing on rotten wood or fallen twigs. *Gerronemamicrocarpum* is mainly distinguished from *G.baishanzuense*, *G.nemorale*, and *G.zhujian* by its distinctly small basidiomata and basidiospores. The tiny brown fur or scales on the pileus of *G.zhujian* differentiate it from the other three species. *Gerronemanemorale* is morphologically most similar to *G.baishanzuense* but can be readily discriminated on the basis of its olive-tinted pileus, larger basidiospores, and smaller caulocystidia.

Significantly, the phylogenetic relationship of *G.subclavatum* to *G.nemorale* remains unresolved given the limited genetic differentiation between these two taxa (Cooper 2014; Latha et al. 2018; Antonín et al. 2019; Vizzini et al. 2019). *Gerronemasubclavatum* was formerly classified as a species in *Omphalina*; the original description is as follows: "Pileus thin, submembranaceous, subclavate or tubaeform, deeply umbilicate, glabrous, grayish brown, 6–12 mm. broad; lamellae subdistant, very decurrent, yellow; stem slender, subpruinose, often tomentose near the base, hollow, whitish, about 2.5 cm. long, 1 mm. thick; spores elliptic, 6–7.5 μm long, 4–5 μm broad" (Peck 1900). A new combination, *G.subclavatum*, was later proposed, but a detailed description was not provided (Singer 1970; Redhead 1986). Taking into account the grayish brown pileus, whitish stipe, and smaller basidiospores of *G.subclavatum*, we believe that this species is morphologically distinct from *G.nemorale*. We therefore tentatively accept *G.subclavatum* and *G.nemorale* as two independent taxa but emphasize that sufficient sampling and a detailed appraisal of the morphological and molecular variation of *G.subclavatum* and *G.nemorale* are required to confirm this hypothesis.

## Supplementary Material

XML Treatment for Gerronema baishanzuense

XML Treatment for Gerronema microcarpum

XML Treatment for Gerronema zhujian

XML Treatment for Gerronema nemorale
